# Using Chatbot Technology to Improve Brazilian Adolescents’ Body Image and Mental Health at Scale: Randomized Controlled Trial

**DOI:** 10.2196/39934

**Published:** 2023-06-19

**Authors:** Emily L Matheson, Harriet G Smith, Ana C S Amaral, Juliana F F Meireles, Mireille C Almeida, Jake Linardon, Matthew Fuller-Tyszkiewicz, Phillippa C Diedrichs

**Affiliations:** 1 Centre for Appearance Research University of the West of England Bristol United Kingdom; 2 Federal Institute of Education, Science and Technology of Southeast of Minas Gerais Barbacena Brazil; 3 Department of Family and Community Medicine, School of Community Medicine University of Oklahoma Health Science Center Tulsa, OK United States; 4 Department of Psychiatry Universidade Federal de São Paulo São Paulo Brazil; 5 School of Psychology Deakin University Geelong, Victoria Australia; 6 Center for Social and Early Emotional Development Deakin University Burwood, Victoria Australia

**Keywords:** adolescent, Brazil, body image, chatbot, microintervention, randomized controlled trial, mobile phone

## Abstract

**Background:**

Accessible, cost-effective, and scalable mental health interventions are limited, particularly in low- and middle-income countries, where disparities between mental health needs and services are greatest. Microinterventions (ie, brief, stand-alone, or digital approaches) aim to provide immediate reprieve and enhancements in mental health states and offer a novel and scalable framework for embedding evidence-based mental health promotion techniques into digital environments. Body image is a global public health issue that increases young peoples’ risk of developing more severe mental and physical health issues. Embedding body image microinterventions into digital environments is one avenue for providing young people with immediate and short-term reprieve and protection from the negative exposure effects associated with social media.

**Objective:**

This 2-armed, fully remote, and preregistered randomized controlled trial assessed the impact of a body image chatbot containing microinterventions on Brazilian adolescents’ state and trait body image and associated well-being outcomes.

**Methods:**

Geographically diverse Brazilian adolescents aged 13-18 years (901/1715, 52.54% girls) were randomized into the chatbot or an assessment-only control condition and completed web-based self-assessments at baseline, immediately after the intervention time frame, and at 1-week and 1-month follow-ups. The primary outcomes were mean change in state (at chatbot entry and at the completion of a microintervention technique) and trait body image (before and after the intervention), with the secondary outcomes being mean change in affect (state and trait) and body image self-efficacy between the assessment time points.

**Results:**

Most participants who entered the chatbot (258/327, 78.9%) completed ≥1 microintervention technique, with participants completing an average of 5 techniques over the 72-hour intervention period. Chatbot users experienced small significant improvements in primary (state: *P*<.001, Cohen *d*=0.30, 95% CI 0.25-0.34; and trait body image: *P*=.02, Cohen *d* range=0.10, 95% CI 0.01-0.18, to 0.26, 95% CI 0.13-0.32) and secondary outcomes across various time points (state: *P*<.001, Cohen *d=*0.28, 95% CI 0.22-0.33; trait positive affect: *P*=.02, Cohen *d* range=0.15, 95% CI 0.03-0.27, to 0.23, 95% CI 0.08-0.37; negative affect: *P*=.03, Cohen *d* range=−0.16, 95% CI −0.30 to −0.02, to −0.18, 95% CI −0.33 to −0.03; and self-efficacy: *P*=.02, Cohen *d* range=0.14, 95% CI 0.03-0.25, to 0.19, 95% CI 0.08-0.32) relative to the control condition. Intervention benefits were moderated by baseline levels of concerns but not by gender.

**Conclusions:**

This is the first large-scale randomized controlled trial assessing a body image chatbot among Brazilian adolescents. Intervention attrition was high (531/858, 61.9%) and reflected the broader digital intervention literature; barriers to engagement were discussed. Meanwhile, the findings support the emerging literature that indicates microinterventions and chatbot technology are acceptable and effective web-based service provisions. This study also offers a blueprint for accessible, cost-effective, and scalable digital approaches that address disparities between health care needs and provisions in low- and middle-income countries.

**Trial Registration:**

Clinicaltrials.gov NCT04825184; http://clinicaltrials.gov/ct2/show/NCT04825184

**International Registered Report Identifier (IRRID):**

RR2-10.1186/s12889-021-12129-1

## Introduction

### Background

Accessible, cost-effective, and scalable mental health interventions are limited, particularly in low- and middle-income countries (LMICs), where disparities between mental health needs and services are greatest [1,2]. Digital interventions offer a unique opportunity to address this gap, particularly among adolescents. For instance, in Brazil, 91% of those aged 9 to 17 years have internet access, with 88% of internet users having a social media profile [3]. Brazilian adolescents use social media for an average of 4 hours per day [4], with most (53.2%) reporting problematic smartphone behaviors including overuse, preoccupation, and withdrawal [5]. Despite this high level of exposure to potentially harmful social media content, digital environments have been underused to connect with and provide Brazilian adolescents with evidence-based mental health and well-being resources, particularly regarding body image.

Body image concerns are a potent risk factor for eating disorders, with this group of mental health concerns incurring a yearly economic cost of US $149 billion in the United States alone [6,7]. Costs are also likely to rise owing to the impact of the global COVID-19 pandemic on body and eating concerns [8]; thus, accessible, cost-effective, and scalable prevention and intervention approaches are critical, particularly for already underserved countries. For instance, body image concerns are a global phenomenon and are reported across various countries and cultures [9]. However, advancements in body image research and the availability of evidence-based prevention and intervention vary greatly between countries. In some countries, body image is an unexplored construct; in others, there are a handful of prevalence studies; or in the case of high-income, English-speaking countries with majority White population, there is an abundance of research on prevalence, causation, prevention, and intervention [10]. This disparity in research does not reflect the prevalence or severity, with body image concerns in LMICs comparable with those in high-income countries [8,10]. Furthermore, owing to political and economic disparities and competing priorities, research and early intervention efforts for body image in LMICs are largely unfunded, unexplored, and unaddressed.

### Previous Work

In Brazil, 8 in 10 young people report body image concerns, with 1 in 5 reporting engagement in disordered eating and unhealthy weight control behaviors [11,12]. However, to date, only 2 body image interventions have been evaluated among Brazilian populations and neither are widely available [13,14]. This is, in part, owing to the intervention modality and the historical and current funding restrictions experienced by Brazilian researchers. First, both interventions involve in-person implementation in group settings; therefore, their sustainability is reliant on human and infrastructural resources. Second, in 2017, the Brazilian government reduced its health budget by US $210 million, with spending cuts of 15% for public universities and 45% for scientific research [15]. Therefore, developing innovative and sustainable evidence-based body image interventions is a challenge for Brazilian researchers. One way to overcome these barriers is to use international partnerships among academia, industry, and community. Such partnerships afford the creation of accessible, cost-effective, and scalable interventions that reach those in need and, in turn, reduce health care disparities.

Chatbot technology offers an innovative pathway for engaging young people with evidence-based resources. In a recent review, 11 mental health chatbots were identified across 12 studies, with the majority targeting affective disorders, including anxiety and depression (eg, *Woebot*) [1]. The chatbots used predefined rules or decision trees (8 out of 12 studies), machine learning, or artificial intelligence (4 out of 12 studies), and half were personified with an avatar. Although the chatbots showed promise as an effective and safe intervention modality, the review heeded caution about their clinical significance relative to treatment as usual and, therefore, concluded that greater research is needed to draw solid conclusions about this emerging service provision. Furthermore, all studies were conducted in high-income countries (eg, Australia, China, Sweden, and the United States), and therefore, the authors called for concerted research efforts in LMICs, stating that there is a greater need for this technology in countries where the shortage of mental health professionals is the highest. To date, no chatbot has been developed for or tested among Brazilians.

*Topity*, a new Brazilian body image chatbot hosted on *Facebook Messenger,* comprises 8 microintervention techniques that address risk and protective factors for body image [16-18]. Microinterventions are generally designed as brief, digital, and self-guided approaches that use in-the-moment techniques to provide immediate symptom relief or enhancement [19]. To date, this intervention model has been predominantly developed for and tested among adult samples with body image and mood concerns, with techniques including brief web-based written tasks and instructional audio and video clips [20,21]. More recently, microinterventions have been developed and applied to young people (eg, short films and web-based games), with these approaches proving acceptable and effective at eliciting immediate and short-term improvements in body image and mood [22,23].

During the *Topity* experience, users are given the choice to interact with either an avatar of a young woman (Dandara) or a man (Gabriel). The chatbot uses predefined rules and gamification to guide users through the completion of 8 microinterventions, which are clustered into 3 themes (*Family, Friends and Body Image* [2 techniques]; *Social Media and Body Image* [4 techniques]; and *Body Appreciation and Functionality* [2 techniques]). The microinterventions were informed by and adapted from existing body image interventions that have been traditionally delivered in hard copy (eg, self-help books) or in-person settings (eg, individual therapy or group-based programs). These techniques teach users how to critically analyze and evaluate social media content to reduce vulnerability to negative influences (ie, media literacy) [16], identify and challenge unhelpful thinking styles and behaviors that perpetuate body image distress (ie, cognitive behavior theory for body image) [17], and appreciate the features and functions of the body beyond appearance (ie, positive body image and embodiment theory) [18]. Each technique takes between 5 and 10 minutes to complete and has a distinct beginning and end. Access to microintervention techniques is gamified, with users needing to complete an initial technique within a thematic cluster before “unlocking” more activities. For example, in the *Family, Friends and Body Image* cluster, users need to complete the *Banish Body Talk* microintervention before being able to access and complete the *Dealing with Provocative People* microintervention. Gamification is a key feature of digital interventions because of its effect on users’ motivation, engagement, and skill mastery [24]. An overview of the cocreation process for *Topity* and a summary of the microintervention techniques are reported in Tables 1 and 2 of the protocol, respectively [25].

*Topity* is 1 of the 4 body- and eating-related chatbots that have been developed in recent years. The other 3 bots, including *KIT* [26], *Tessa* [27], and *Alex* [28], target English-speaking populations and offer different service provisions. Furthermore, each chatbot is at a different phase of the development or testing process (eg, user experience vs effectiveness). First, *KIT* addresses body and eating concerns by providing users with psychoeducation, help seeking, and coping strategies. It was highly acceptable among users in Australia, and its effectiveness has been reported in gray literature [26]. Second, *Tessa* features the *Body Positive* program, which is a distilled version of the cognitive-behavioral therapy web-based program, *StudentBodies*, and comprises 8- × 10-minute conversations with the chatbot [27]. *Body Positive* has proven effective at reducing weight and shape concerns among at-risk American women (mean age 21, SD 3.09 years) and shows potential for reducing the onset of eating disorders. Finally, and most recently, *Alex* was developed to increase treatment motivation and the use of mental health services among individuals who screen positive for an eating disorder and are not yet in treatment [28]. *Alex* is currently undergoing efficacy testing [29].

### Our Study

This 2-armed, fully remote randomized controlled trial (RCT) addresses several gaps within the limited but emerging field of mental health chatbots. It is the first body image chatbot for non–English-speaking users and one of the first studies to assess a chatbot in an LMIC [1]. Specifically, this trial evaluated the effectiveness of *Topity* in eliciting immediate and short-term improvements in adolescents’ state- and trait-based body image, affect, and self-efficacy in managing body image concerns. Research hypotheses were prespecified on page 3 of the protocol [25] and were formulated for overall chatbot efficacy (hypotheses 1-2), moderation effects within subsamples (hypothesis 3), and intervention engagement and adherence (hypothesis 4):

1. *Hypothesis 1:* the chatbot was designed to provide immediate benefits to users. Therefore, it is anticipated that adolescents will experience improvements in state-based body satisfaction and affect at the time of engaging with the chatbot.
2. *Hypothesis 2:* adolescents randomized into the intervention condition will experience greater improvements in trait-based body esteem, affect, and body image self-efficacy immediately after the intervention time frame (eg, postintervention assessment) and at the 1-week and 1-month follow-ups relative to the assessment-only control condition.
3. *Hypothesis 3:* on the basis of previous research on the moderating effects of gender [30] and trait psychopathology [23,31] on body image intervention effectiveness, it was hypothesized that intervention effects will be moderated by gender and baseline levels of body esteem, affect, and body image self-efficacy. Specifically, intervention effects will be greatest among girls, girls and boys with lower levels of trait body esteem and body image self-efficacy, and girls and boys with higher levels of trait negative affect.
4. *Hypothesis 4:* with regard to user engagement and adherence, given the novelty of this intervention, analyses will be exploratory. However, it is anticipated that greater engagement and adherence with chatbot interventions will lead to greater improvements in trait-based outcomes.

## Methods

### Study Design

The study was a 2-armed, fully remote, and preregistered RCT conducted between April 7 and August 8, 2021, in Brazil. Details of the study rationale and protocol have been published elsewhere [25]. The research was conducted in accordance with ethical standards and guidelines for conducting research on young people in Brazil. Before obtaining consent and assent, parents and participants were provided with an information sheet outlining the research procedures and associated risks, respectively. Consent withdrawal was possible at any time without cause for justification. At study completion, participants were provided with a debrief form disclosing research aims, ancillary mental health resources and an electronic voucher of R$100 (approximately US $20) and R$80 (approximately US $15) for the intervention and control conditions, respectively. Data were anonymized and accessed only by the authoring research team using masked and password-protected data files.

### Ethics Approval and Trial Registration

This study received ethics approval from the *Instituto Federal de Educação, Ciência e Tecnologia do Sudeste de Minas Gerais* (4.232.804); *Comissão Nacional de Ética em Pesquisa* (4.582.466); and the University of the West of England (HAS 19.12.090). The study was registered with ClinicalTrials.gov (NCT04825184) [32].

### Participants

The participants were recruited from diverse ethnic, geographic, and socioeconomic backgrounds across Brazil. Recruitment was conducted via a Brazilian research agency (ie, via email to their participant databases) and United Nations International Children’s Emergency Fund’s web-based communication platforms (eg, U-Report; a free tool for community participation). Eligible participants were adolescents aged 13 to 18 years who spoke Brazilian Portuguese, were a Brazilian resident, and had access to *Facebook Messenger*.

### Randomization and Masking

Participants were randomly assigned 1:1 to receive either the chatbot intervention or an assessment-only control. The randomization scheme was generated by a research agency using a validated computer software. Blinding of the participants was not possible because of the nature of the intervention. The risk of bias from researchers was minimized because of having no contact with participants during the trial (ie, recruitment; randomization, survey dissemination, and compensation was completed by the research agency). Data analysts were blinded when conducting analyses of primary and secondary trait outcomes; however, this was not possible for primary and secondary state measures because of the within-group design.

### Procedure

**Figure 1:**
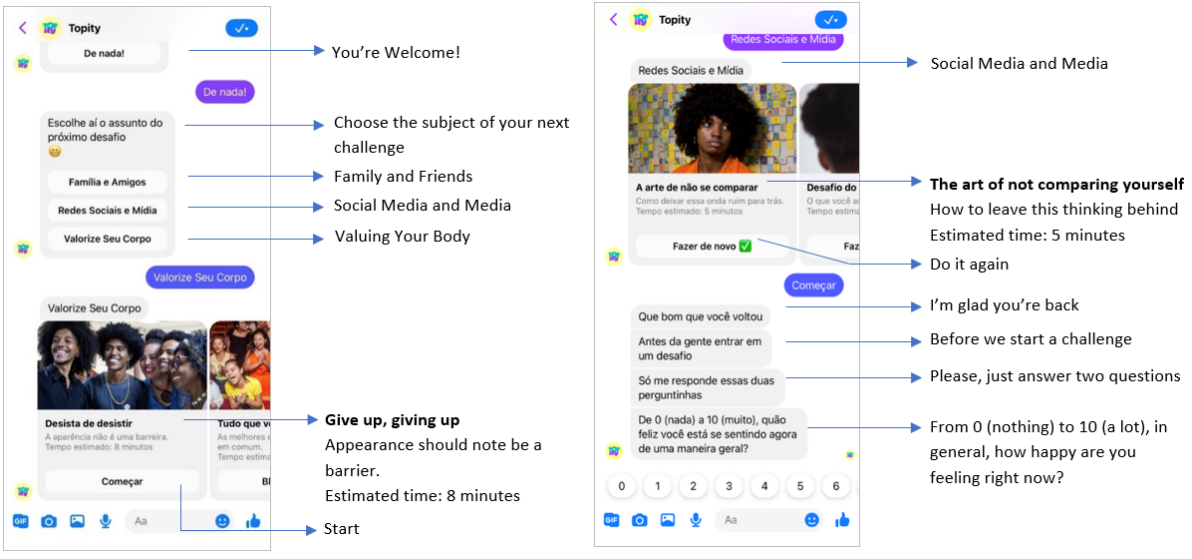
Screenshots and translations of a Topity conversation on mobile view.

Following parental consent and participant assent, eligible participants completed preintervention self-assessments via a closed web-based survey (received via email), after which they were randomized into the intervention or control conditions. The web-based survey underwent user testing before the trial began and the research agency monitored the response rate and completion. Those in the intervention condition received a unique access link to the chatbot, 24 hours after randomization, and were encouraged to interact with the intervention as much as possible over the next 72-hour period (Figure 1). The unique access code was only compatible with the assigned user’s *Facebook Messenger*, thus ensuring confidential interactions between the user and chatbot and prohibiting nonparticipants from entering the chatbot during the trial period. The control condition received “standard online care” for body image concerns among Brazilian adolescents, which at present is no intervention. Initially, *Topity* users had access to 3 of the 8 microintervention techniques (eg, the first technique from each thematic cluster), and once completed, they gained access to the next technique in the cluster. Therefore, once a technique was unlocked, there were no limits to the number of completions. The ordering of techniques was informed by the difficulty of the microintervention concept and skill, whereby users mastered easier concepts and skills (eg, psychoeducation) before progressing to those that were more challenging (eg, behavior and cognition change). Upon entering the chatbot and after completing a microintervention technique, users were assessed for state body satisfaction and affect. In the event that participants did not engage with the bot after 12, 16, and 23.5 hours, they received a *Facebook Messenger* notification encouraging participation. At the completion of the 72-hour intervention period, all participants were sent postintervention surveys and again at the 1-week and 1-month time points. During the 1-month follow-up period, all participants were provided with contact details for accessible mental health support services in Brazil, and the control condition was invited to interact with *Topity*; however, their engagement was not monitored or assessed for effectiveness. The participants received an electronic voucher of R$100 (approximately US $20) and R$80 (approximately US $15) for the intervention and control condition, respectively. Although participants were aware that they would receive compensation at study commencement, the compensation amount was not disclosed until study completion.

### Outcomes

A comprehensive overview of the outcome measures is provided in Table 3 of the protocol [25]. The primary outcome measures were the mean change in state (ie, a single 11-point scale) and trait (ie, Body Esteem Scale for Adolescents and Adults Brazil [33]) body image—specifically, the mean change in state body satisfaction between chatbot entry and at the completion of a microintervention technique and the mean change in trait body esteem between preintervention and postintervention assessments. Secondary outcomes included the mean change in state affect (ie, a single 11-point scale), trait positive and negative affect (ie, the Positive and Negative Affect Scale for Children 8-item [34]), and self-efficacy in addressing body image concerns (ie, The Body Image Self-Efficacy Scale [35-37]). Treatment adherence (eg, ≥1 microintervention technique) and user acceptability were also assessed after the intervention time frame.

### Statistical Analysis

Statistical analyses were prespecified on pages 10 and 11 of the protocol [25]. Specifically, missing data were handled using multiple imputations (m=50) with chained equations, and participants were retained in the group they were assigned to at baseline, consistent with the principles of intention-to-treat. Linear mixed models were used to test our hypotheses. For hypothesis 1, scores on state-based outcomes were regressed onto a time variable (coded 0=precontent; 1=postcontent) for the intervention arm only. For hypotheses 2 to 4, trait-level outcome variables were regressed onto a time variable (dummy coded as baseline vs after the intervention, 1-week follow-up, and 1-month follow-up), group (0=control; 1=intervention), and an interaction between time and group to evaluate efficacy. Random effects were included for time and an unstructured covariance matrix was used to estimate the covariance among these random effects. Hypotheses 3 and 4 included the moderators of these random effects over time.

### Protocol Amendments

Owing to the recruitment processes being heavily reliant on parents’ literacy levels to provide informed consent, a video communicating the study information was embedded into the recruitment materials. This amendment was implemented 2 weeks into recruitment, following feedback from stakeholders that the original recruitment methods were restrictive. Furthermore, the recruitment phase was extended from 2 to 12 weeks because of slow uptake. Extensions were incrementally increased as part of the review process (eg, every 2 weeks). This rate was somewhat accelerated by adapting the recruitment materials; however, given that the trial was conducted during the peak of the COVID-19 pandemic in Brazil, there are likely several contextual factors outside the researchers’ control that led to lower than anticipated uptake (eg, screen and social media fatigue) [38].

## Results

### Baseline Characteristics

The baseline characteristics of the participants are presented in Table 1. There was an approximately equal number of girls and boys and a nationally representative distribution across ethnicity and region. There were no notable differences and negligible effect sizes on any baseline variables between the intervention and control groups, indicating that the randomization process was successful.

**Table 1:** Baseline characteristics of the sample.

Variable^a^			Total sample (N=1715)		Intervention (n=858)		Control (n=857)		*P* value			ES^b^	
**Age (years), n (%)**										.29			0.06
	13	276 (16.1)		135 (15.7)		141 (16.5)							
	14	237 (13.8)		108 (12.6)		129 (15.1)							
	15	230 (13.4)		124 (14.5)		106 (12.4)							
	16	255 (14.9)		140 (16.3)		115 (13.4)							
	17	220 (12.8)		109 (12.7)		11 (1.3)							
	18	497 (29)		242 (28.2)		255 (29.8)							
**Gender, n (%)**										.64			0.02
	Boy	801 (46.7)		406 (47.3)		395 (46.1)							
	Girl	901 (52.5)		447 (52.1)		454 (53)							
	Gender diverse	13 (0.8)		5 (0.6)		8 (0.9)							
**Ethnicity, n (%)**										.91			0.03
	Asian	40 (2.3)		23 (2.7)		17 (2)							
	Black	181 (10.6)		92 (10.7)		89 (10.4)							
	Indigenous	14 (0.8)		8 (0.9)		6 (0.7)							
	Mixed race	630 (36.7)		314 (36.6)		316 (36.9)							
	White	845 (49.3)		419 (48.8)		426 (49.7)							
	Other	5 (0.3)		2 (0.2)		3 (0.4)							
**Region, n (%)**										.68			0.03
	North	92 (5.4)		50 (5.8)		42 (4.9)							
	Northeast	348 (20.3)		176 (20.5)		172 (20.1)							
	Central west	134 (7.8)		60 (7)		74 (8.6)							
	Southeast	906 (52.8)		452 (52.7)		454 (53)							
	South	235 (13.7)		120 (14)		115 (13.4)							
**Baseline variables, mean (SD)**													
	Appearance positive	3.33 (0.83)		3.36 (0.84)		3.31 (0.83)		.24			0.05		
	Appearance negative	3.34 (1.08)		3.36 (1.08)		3.33 (1.08)		.64			0.02		
	Weight	3.11 (1.18)		3.10 (1.19)		3.12 (1.17)		.70			0.01		
	Positive affect	3.55 (0.96)		3.56 (0.97)		3.54 (0.96)		.58			0.02		
	Negative affect	2.52 (0.98)		2.50 (0.96)		2.54 (0.99)		.37			0.04		
	Body image self-efficacy	64.73 (22.18)		65.71 (22.04)		63.75 (22.28)		.07			0.08		

^a^Test statistic: chi-square for nominal variables and 2-tailed *t* tests for continuous variables.

^b^ES: effect size (phi coefficient for nominal variables and Cohen *d* for continuous variables).

### Attrition, Adherence, and Acceptability

**Figure 2:**
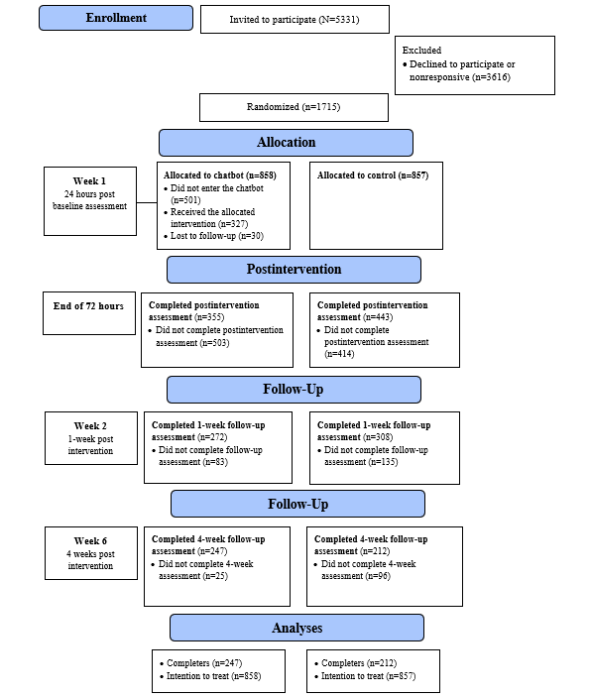
Research design and participant flow using CONSORT (Consolidated Standards of Reporting Trials) eHealth guidelines.

The participant flow diagram is shown in Figure 2. Of the 1715 participants, 798 (46.53%) provided postintervention data, 580 (33.82%) provided 1-week follow-up data, and 459 (26.76%) provided 1-month follow-up data. The intervention group had significantly higher drop-out rates at the postintervention assessment point, relative to the control condition (503/858, 58.6% vs 414/857, 48.3%; *P*<.001; However, the study condition was not significantly associated with dropout at 1 week (586/858, 68.3% vs 579/857, 64.1%) or 1 month (611/858, 71.2% vs 645/857, 75.3%) follow-up (*P* values >.05). Participants who dropped out at the primary time point (after the intervention time frame) were compared with those who completed the baseline variables (Table 2). Dropouts reported significantly lower weight esteem scores at baseline than completers. Notable differences also existed in age (those aged 18 years were the most likely to drop out), ethnicity (Indigenous participants were the most likely to drop out), and region (those from the central west region were the least likely to drop out).

Of the 858 participants randomized into the intervention group, 327 (38.1%) entered the chatbot, and of those 327 participants, 258 (78.9%) completed 1 or more microintervention techniques, thus meeting treatment adherence. On average, participants completed 5 techniques over the 72-hour intervention period, with a minimum of 1 and a maximum of 17 completed across participants. Finally, most participants (251/327, 77%) selected Dandara as their avatar, and 155 of the postintervention responses included acceptability data, with *Topity* receiving an overall score of 6.07 out of 7.

**Table 2:** Comparisons of dropouts and completers after the intervention time frame on baseline variables.

Variables^a^		Dropout T2^b^	Completer T2	*P* value		ES^c^	
**Gender, n (%)**					.32		0.03
	Boy (n=801)	442 (55.2)	359 (44.8)				
	Girl (n=901)	467 (51.8)	434 (48.2)				
	Gender diverse (n=13)	8 (61.5)	5 (38.5)				
**Age (years), n (%)**					<.001		0.30
	13 (n=276)	118^d^ (42.8)	158^d^ (57.2)				
	14 (n=237)	107^d^ (45.1)	130^d^ (54.9)				
	15 (n=230)	93^d^ (40.4)	137^d^ (59.6)				
	16 (n=255)	115^d^ (45.1)	140^d^ (54.9)				
	17 (n=220)	102^d^ (46.4)	118^d^ (53.6)				
	18 (n=497)	382^d^ (76.9)	115^d^ (23.1)				
**Ethnicity, n (%)**					.04		0.08
	Asian (n=40)	23 (57.5)	17 (42.5)				
	Black (n=181)	99 (54.7)	82 (45.3)				
	Indigenous (n=14)	12^d^ (85.7)	2^d^ (14.3)				
	Mixed race (n=630)	355 (56.3)	275 (43.7)				
	White (n=845)	426^d^ (50.4)	419^d^ (49.6)				
	Other (n=5)	2 (40)	3 (60)				
**Region, n (%)**					.005		0.09
	North (n=92)	57 (62)	35 (38)				
	Northeast (n=348)	202 (58)	146 (42)				
	Central west (n=134)	58^d^ (43.3)	76^d^ (56.7)				
	Southeast (n=906)	464^d^ (51.2)	442^d^ (48.8)				
	South (n=235)	136 (57.9)	99 (42.1)				
**Baseline, mean (SD)**							
	Appearance positive	3.31 (0.81)	3.36 (0.86)	.22		0.05	
	Appearance negative	3.37 (1.07)	3.31 (1.08)	.20		0.05	
	Weight	3.03 (1.17)	3.20 (1.18)	.003		0.14	
	Positive affect	3.57 (0.98)	3.54 (0.95)	.52		0.03	
	Negative affect	2.54 (0.98)	2.50 (0.98)	.41		0.04	
	Body image self-efficacy	64.32 (22.15)	65.21 (22.21)	.41		0.04	

^a^Test statistic: chi-square for nominal variables and 2-tailed *t* tests for continuous variables.

^b^T2 refers to the postintervention time point.

^c^ES: effect size (phi coefficient for nominal variables and Cohen *d* for continuous variables).

^d^When groups significantly differed in a pairwise Bonferroni corrected significance test.

### Main Statistical Analyses

#### Hypothesis 1

Hypothesis one tested whether engagement with the chatbot produced immediate increases in the state of body satisfaction and positive affect. The results revealed significant main effects of time on satisfaction (unstandardized coefficient, *b*=0.60, 95% CI 0.50-0.70; *P*<.001; Cohen *d*=0.30) and affect (*b*=0.51, 95% CI 0.41-0.61; *P*<.001; Cohen *d*=0.28), indicating that participants reported momentary improvements in these outcomes immediately following exposure to chatbot microintervention techniques.

#### Hypothesis 2

##### Overview

Hypothesis 2 tested the differences in primary and secondary trait outcomes immediately after the intervention and at the 2 follow-up points. The results of these analyses are presented in Table 3.

**Table 3:** Means, SDs, and change scores on outcomes between study groups.

Outcomes				Study group										Difference in change score			Cohen *d*			*P* value (2-tailed)
				Control					Experimental					Experimental-control^a^						
				Values, n		Values, mean (SD)^b^		Values, n			Values, mean (SD)		Values, mean (95% CI)							
**Primary outcome**																				
	**Appearance positive**																			
		Baseline	857		3.31 (0.83)		858			3.36 (0.85)		N/A^c^			N/A			N/A		
		Postintervention	443		3.32 (0.86)		355			3.52 (0.84)		0.11 (0.02 to 0.19)			0.13			.02		
		1-week follow-up	308		3.39 (0.88)		272			3.67 (0.85)		0.09 (0.01 to 0.18)			0.1			.03		
		1-month follow-up	246		3.35 (0.88)		213			3.74 (0.81)		0.22 (0.13 to 0.32)			0.26			<.001		
	**Appearance negative**																			
		Baseline	857		3.34 (1.09)		858			3.36 (1.08)		N/A			N/A			N/A		
		Postintervention	443		3.29 (1.09)		355			3.46 (1.07)		0.12 (0.00 to 0.24)			0.11			.047		
		1-week follow-up	308		3.31 (1.11)		272			3.58 (1.12)		0.15 (0.04 to 0.27)			0.13			.01		
		1-month follow-up	246		3.32 (1.11)		213			3.6 (1.03)		0.15 (0.02 to 0.28)			0.13			.02		
	**Weight**																			
		Baseline	857		3.12 (1.17)		858			3.1 (1.19)		N/A			N/A			N/A		
		Postintervention	443		3.23 (1.12)		355			3.35 (1.1)		0.02 (−0.10 to 0.13)			0.01			.70		
		1-week follow-up	308		3.28 (1.1)		272			3.54 (1.12)		0.1 (−0.02 to 0.22)			0.08			.10		
		1-month follow-up	246		3.33 (1.1)		213			3.65 (1.02)		0.19 (0.07 to 0.31)			0.16			.003		
**Secondary outcome**																				
	**Positive affect**																			
		Baseline	857		3.54 (0.96)		858			3.57 (0.98)		N/A			N/A			N/A		
		Postintervention	443		3.55 (0.94)		355			3.72 (0.93)		0.09 (−0.03 to 0.19)			0.09			.17		
		1-week follow-up	308		3.56 (0.98)		272			3.82 (1.01)		0.15 (0.03 to 0.27)			0.15			.02		
		1-month follow-up	246		3.6 (0.96)		213			3.93 (0.91)		0.23 (0.08 to 0.37)			0.23			.002		
	**Negative affect**																			
		Baseline	857		2.54 (0.99)		858			2.5 (0.97)		N/A			N/A			N/A		
		Postintervention	443		2.45 (1.01)		355			2.34 (1.03)		−0.07 (−0.20 to 0.07)			−0.07			.30		
		1-week follow-up	308		2.42 (1.06)		272			2.23 (1.05)		−0.16 (−0.30 to −0.02)			−0.16			.03		
		1-month follow-up	246		2.37 (1.02)		213			2.15 (1.01)		−0.18 (−0.33 to −0.03)			−0.18			.02		
	**Body image self-efficacy**																			
		Baseline	857		63.76 (22.28)		858			65.72 (22.05)		N/A			N/A			N/A		
		Postintervention	443		65.92 (22.87)		355			70.44 (21.7)		2.18 (−0.54 to 4.90)			0.09			.12		
		1-week follow-up	308		67.27 (23.36)		272			73.37 (21.41)		3.13 (0.63 to 5.63)			0.14			.02		
		1-month follow-up	246		67.83 (24.29)		213			76.43 (20.21)		4.37 (1.70 to 7.04)			0.19			.002		

^a^Experimental-control: calculation of the mean difference between groups by subtracting the control group's mean from the experimental group's mean.

^b^Mean (SD) values are based on nonimputed data; mean differences and effect sizes were derived from the intention-to-treat analysis.

^c^N/A: not applicable.

##### After the Intervention Time Frame

For the primary outcome of body esteem, significant mean differences were observed for the appearance positive (Cohen *d*=0.13; *P*=.02) and negative (Cohen *d*=0.11; *P*=.047) subscales, in favor of the intervention group experiencing improvements. A significant mean difference was not observed for the weight subscale (Cohen *d*=0.01; *P*=.75). Nonsignificant mean differences between the 2 groups immediately after the intervention were also observed for the secondary outcomes of positive affect (Cohen *d*=0.09), negative affect (Cohen *d*=−0.07), and body image self-efficacy (Cohen *d*=0.14).

##### Follow-Up

As seen in Table 3, significant mean differences were observed at both the 1-week and 1-month follow-up on all primary and secondary outcomes, except for the weight subscale at the 1-week follow-up. In all cases, the intervention group experienced greater improvements in these constructs than the control group. Effect sizes were small, ranging from Cohen *d*=0.10 to Cohen *d*=0.26.

#### Hypothesis 3

Hypothesis 3 tested whether mean differences in trait outcomes at each time point were moderated by participant gender and baseline severity. The results of these analyses are shown in Tables S1 and S2 in Multimedia Appendix 1.

Participant gender was not a notable moderator for any outcome variable immediately after the intervention time frame or at any of the 2 follow-up periods. However, there is some evidence to suggest that baseline levels of a particular outcome significantly affect responsiveness to the intervention. In particular, those with lower body esteem (on all 3 subscales), lower positive affect, and lower body image self-efficacy experienced greater intervention benefits than those with higher levels on these outcome variables. This occurred at all 3 time points for the appearance positive subscale, at the 1-week and 1-month follow-ups for the appearance negative and weight subscales and body image self-efficacy, and only at the 1-month follow-up for positive affect.

#### Hypothesis 4

Hypothesis 4 tested whether there were any relationships between the 4 indices of chatbot use and the level of improvement in the primary and secondary outcomes. The results of these analyses are presented in Table S3 in Multimedia Appendix 1. We did not observe any notable relationships between the levels of chatbot use and the primary and secondary outcomes immediately after the intervention time frame and follow-up periods.

## Discussion

### Principal Findings

This 2-armed, fully remote RCT was the first to assess body image chatbot among Brazilian adolescents. These findings indicate that a chatbot containing microinterventions is an effective approach for eliciting small notable improvements in state- and trait-based outcomes for body image and associated well-being constructs. Girls and boys aged 13 to 18 years experienced small but substantial improvements in state body satisfaction and positive affect immediately following engagement with *Topity* techniques. Users also reported small notable improvements in trait-based body esteem, positive and negative affect, and body image self-efficacy relative to the assessment-only control condition. These group differences were observed at all 3 time points for the appearance positive and negative subscales of the Body Esteem Scale for Adolescents and Adults, and at 1-week and 1-month follow-ups for positive and negative affect and body image self-efficacy. Group differences in the weight subscale of the Body Esteem Scale for Adolescents and Adults were only observed at 1-month, in favor of the intervention group experiencing improvements. The intervention effects were comparable across girls and boys; therefore, they were not moderated by gender. However, the effects were moderated by baseline concerns, with those reporting lower baseline body esteem, positive affect, and self-efficacy experiencing greater intervention benefits than those with lower levels of concern. Finally, no dose effects were observed (eg, greater engagement was not associated with greater improvement).

### Comparison With Previous Work

This is the third RCT to report small immediate and sustained improvements in young people’s state- and trait-based body image following engagement with a microintervention [22,23]. These small but notable effects across all 3 trials are likely owing to both the brevity of the intervention phases (ie, single sessions; 72 hours) and the use of universal samples (ie, varying degrees of body image concerns). Larger and more sustained effects have been observed in microintervention [39] and chatbot [27] studies with longer intervention phases (ie, 3 weeks [39]; 1 month [27]) and selected samples (eg, women with heighted weight and shape concerns), which is likely owing to the greater scope for symptom change. The current moderator analyses mirror these selected sample effects, with intervention benefits being the largest among young people with poor baseline levels of body image, affect, and self-efficacy. Notably, improvements in secondary outcomes (ie, positive and negative affect and body image self-efficacy) were not observed immediately after the intervention but emerged at 1-week and 1-month follow-ups. This pattern may be attributed to the specificity of the intervention content and mediating effect of improved body image on affect and self-efficacy, that is, body image has shown to be predictive of mood states [40], and participants may need to experience improved body image before thinking and feeling that they are self-efficacious in managing their body image concerns.

With respect to intervention adherence and user engagement, less than half (327/858, 44% of participants) of those randomized into the intervention group used the chatbot. Of these, the majority (258/327, 79% of participants) completed the minimum intervention dose of one microintervention technique, with an average of 5 techniques completed over the 72-hour intervention period. There was also a preference for the woman avatar, Dandara, with most girls and boys opting to receive guidance from her, relative to her counterpart, Gabriel. These engagement levels [27,39] and gender preferences [41] mirror previous body image research.

First, with respect to engagement levels, in 2 comparable microintervention [39] and chatbot [27] trials, women completed an average of 4 techniques over 21-day and 1-month periods, respectively. Although the number of techniques is comparable across the 3 trials, the current participants completed more techniques over a shorter period. More research is needed on the naturalistic and longitudinal use of microinterventions and chatbots to better understand how and when users engage with the content, particularly when an intervention time frame is not prescribed. Relatedly, this trial did not find support for dose response, with intervention effects unaffected by the number of techniques completed. To our knowledge, only one other microintervention study has considered dose response and found no support for this relationship [42].

Second, with respect to gender preferences of the avatar, a small body of research has examined the role of gender in body image interventions, particularly the gender of the intervention facilitator [41]. Specifically, adult women preferred when interventions were delivered by a woman, whereas men did not have a preference and were content with either a man or a woman. The current findings reflect this pattern among adolescents, with most girls (152/174, 87%) selecting Dandara and boys selecting a combination of Dandara (94/147, 64%) and Gabriel (53/147, 36%). Overall, there is limited research on adolescents’ chatbot intervention preferences, including avatar demographics (eg, age, gender, sexuality, and ethnicity), hosting platforms (eg, Facebook and WhatsApp), and chat functions (eg, predefined rules, machine learning, or artificial intelligence). These features require exploration with the end user during intervention development, to ensure an acceptable, feasible, and safe intervention is created.

The current attrition rates and patterns are also comparable with the abovementioned trials [27,39] and broader digital mental health research [43]. First, with respect to condition-specific dropouts, rates were higher in the current intervention condition relative to the control, a pattern that is likely owing to higher participant burden among this group. Second, with respect to overall attrition, rates were higher but still reflective of digital mental health interventions, including body- and eating-related interventions [44] and chatbots [45]. The current rates are likely explained by several methodological features known to exacerbate attrition in digital trials, including a burdensome recruitment and onboarding process, no contact or follow-up calls with a researcher, and the masking of the compensation amount [43]. These features are discussed in this section.

First, Brazil passed the General Data Protection Law in 2018, with policies coming into effect in February 2020. These laws require parents to read, sign, and upload information and consent forms to a secure portal for identity verification before their child’s participation. This process requires parents to have good literacy skills, and without literacy skills, they are unable to engage with and comprehend the research materials, thus restricting adolescents’ participation. The recruitment methods were adjusted based on stakeholder feedback, with an information video provided alongside the written content. However, parents were still required to sign and upload consent forms. Second, to streamline the recruitment and onboarding processes, communication with participants was conducted primarily through automated processes (eg, email), with little to no contact between participants and the researchers or a recruitment agency. Third, during the informed consent phase, participants were advised that they would receive compensation at study completion; however, according to Brazilian ethics, the amount was not disclosed until study completion (eg, at the 1-month follow-up). Collectively, these methodological features and the general nature of digital interventions likely explain the current attrition rates.

Overall, previous and present findings indicate that microinterventions are an effective intervention for young people; however, they may serve different purposes depending on the severity of a participant’s concern. From a stepped-care approach, microinterventions may serve as a stand-alone approach for young people with milder concerns (eg, offered to young people during a moment of body image distress) or as an adjunctive approach to those participating in longer traditional body image interventions (eg, offered to young people ahead of talking therapy to increase motivation and self-efficacy). Unfortunately, to date, a stepped-care model for the eHealth of body and eating concerns has not been formally conceptualized and warrants consideration. Finally, given the growing literature on microinterventions, a systematic review and meta-analysis of this intervention model are both timely and necessary. Specifically, the review should provide a comprehensive overview of extant mental health microinterventions and analyze different intervention and trial features to determine those associated with greater engagement, adherence, and effectiveness.

### Limitations, Strengths, and Future Impact

This trial is not without limitations, most of which speak to the digital and remote nature of the trial, which were compounded by the global COVID-19 pandemic. As noted, this trial comprised several methodological features known to impact attrition (eg, a burdensome recruitment and onboarding process, no contact or follow-up calls with a researcher; the masking of the compensation amount). Furthermore, the trial was conducted during the peak of the COVID-19 pandemic in Brazil (ie, 92,625 cases per day) [46]. Notably, a high level of screen and social media fatigue was reported among adolescents during the pandemic [38], which is largely because of the exponential reliance on technology during this socially restrictive period (ie, web-based schooling and entertainment [eg, social media and streaming services] and video calls with friends and family). Moreover, numerous web-based mental health resources were developed, tested, or made available to young people during this time. Therefore, although body and eating concerns were equally problematic during the pandemic [8], it is possible that this intervention and research opportunity were dismissed because of the overwhelming digital demands of adolescents during this time. Finally, dropout was affected by participants’ age (those aged 18 years were the most likely to drop out), ethnicity (Indigenous participants were the most likely to drop out), and region (those from the central west region were the least likely to drop out). This suggests poor acceptance among these demographics and warrants further exploration.

Despite these limitations, the strengths and findings associated with this trial provide avenues for advancing this research field beyond those previously discussed. First, *Topity* has reached >40,000 young people since the RCT began in April 2021. Second, the digital infrastructure developed for this chatbot can be implemented and disseminated in other countries, with the content adapted for suitability in these contexts. This may include translating the content into different languages and incorporating intervention stimuli that are appropriate and salient to a particular country and culture. Next, this trial provides further support for the adaptation of traditional prevention and intervention techniques (eg, hard copy and in-person) for use in digital environments [47]. More broadly, mental health researchers are encouraged to examine existing evidence-based approaches and identify techniques that could be adapted for stand-alone use in digital environments. Isolating these techniques for self-guided use in digital settings that are already frequented by the consumer is likely to increase the accessibility, acceptability, and scalability of mental health resources and, in turn, lead to impactful and sustainable change.

### Conclusions

This trial supports the use of chatbot technology to deliver mental health microinterventions within digital environments frequented by young people (eg, social media platforms and messaging apps). Microinterventions are proving to be an effective method for providing adolescents with immediate and short-term symptom relief and reducing imbalances in the ratio of harmful and helpful body image content on social media platforms. Although microinterventions are a promising intervention model, more research is needed to conceptualize how this model can be integrated into and enhance a stepped-care model for digital approaches targeting body and eating concerns.

## Appendix

Multimedia Appendix 1 Supplementary tables pertaining to results of hypotheses 3 and 4.

Multimedia Appendix 2 CONSORT E-HEALTH Checklist (V 1.6.1).

## Abbreviations

LMIC: low- and middle-income country
RCT: randomized controlled trial

## Data Availability

The body image chatbot, *Topity*, is freely available to those with access to *Facebook Messenger*. Anonymized survey data will be available on reasonable request for noncommercial purposes.
