# Supplementary material for: Using Chatbot Technology to Improve Brazilian Adolescents’ Body Image and Mental Health at Scale: Randomized Controlled Trial
Source: JMIR Mhealth Uhealth. 2023 Jun 19;11:e39934. doi: 10.2196/39934 (PMC10337468; doi:10.2196/39934)
Supplement: Multimedia Appendix 1 [file mhealth_v11i1e39934_app1.docx]

This is a Multimedia Appendix to a full manuscript published in the J Med Internet Res. For full copyright and citation information see <http://dx.doi.org/10.2196/jmir.xxxx> (to be added after publication)

| Table S1  Analyses Testing whether Gender Moderates Intervention Effects | | | | | | | | | | | | | | | | | | | | | | | |
| --- | --- | --- | --- | --- | --- | --- | --- | --- | --- | --- | --- | --- | --- | --- | --- | --- | --- | --- | --- | --- | --- | --- | --- |
|  | | | **Difference in change score** | | | | | |  | |  | | |  | | |  |  | | |  | | |
|  | | | **(Exp – control )** | | | | | | **x Gender (boy vs girl)** | | | | | | | | **x Gender (boy vs other)** | | | | | | |
| **Outcome** | | | **Mean** | | **95% CIs** | | **p (two-tailed)** | | **b** | | **95% CIs** | | | **p (two-tailed)** | | | **b** | **95% CIs** | | | **p (two-tailed)** | | |
| Appearance positive | | |  | |  | |  | |  | |  | | |  | | |  |  | | |  | | |
| Post-intervention | | | 0.1 | | -0.02, 0.22 | | 0.105 | | 0.01 | | -0.14, 0.16 | | | 0.904 | | | 0.26 | -0.71, 1.23 | | | 0.601 | | |
| 1-week follow-up | | | 0.09 | | -0.02, 0.21 | | 0.103 | | 0 | | -0.14, 0.13 | | | 0.961 | | | 0.13 | -0.10, 0.14 | | | 0.758 | | |
| 1-month follow-up | | | 0.22 | | 0.10, 0.33 | | <.001 | | 0.02 | | -0.10, 0.14 | | | 0.77 | | | -0.08 | -0.82, 0.66 | | | 0.84 | | |
| Appearance negative | | |  | |  | |  | |  | |  | | |  | | |  |  | | |  | | |
| Post-intervention | | | 0.1 | | -0.06, 0.26 | | 0.232 | | 0.04 | | -0.17, 0.25 | | | 0.725 | | | 0.29 | -1.03, 1.62 | | | 0.663 | | |
| 1-week follow-up | | | 0.1 | | -0.05, 0.26 | | 0.188 | | 0.1 | | -0.09, 0.28 | | | 0.313 | | | 0.27 | -0.88, 1.42 | | | 0.648 | | |
| 1-month follow-up | | | 0.11 | | -0.04, 0.27 | | 0.159 | | 0.07 | | -0.09, 0.24 | | | 0.38 | | | -0.03 | -1.04, 0.98 | | | 0.953 | | |
| Weight | | |  | |  | |  | |  | |  | | |  | | |  |  | | |  | | |
| Post-intervention | | | 0.01 | | -0.15, 0.16 | | 0.943 | | 0.03 | | -0.17, 0.23 | | | 0.781 | | | 0.02 | -1.24, 1.28 | | | 0.978 | | |
| 1-week follow-up | | | 0.04 | | -0.11, 0.20 | | 0.587 | | 0.11 | | -0.08, 0.31 | | | 0.256 | | | -0.13 | -1.35, 1.09 | | | 0.834 | | |
| 1-month follow-up | | | 0.13 | | -0.02, 0.28 | | 0.093 | | 0.12 | | -0.04, 0.28 | | | 0.154 | | | -0.21 | -0.04, 0.28 | | | 0.678 | | |
| Positive affect | | |  | |  | |  | |  | |  | | |  | | |  |  | | |  | | |
| Post-intervention | | | 0.05 | | -0.11, 0.21 | | 0.519 | | 0.05 | | -0.15, 0.26 | | | 0.62 | | | 0.38 | -0.90, 1.66 | | | 0.563 | | |
| 1-week follow-up | | | 0.12 | | -0.05, 0.28 | | 0.156 | | 0.06 | | -0.13, 0.26 | | | 0.533 | | | 0.03 | -1.20, 1.25 | | | 0.967 | | |
| 1-month follow-up | | | 0.19 | | 0.01, 0.37 | | 0.037 | | 0.08 | | -0.11, 0.27 | | | 0.425 | | | -0.05 | -1.17, 1.08 | | | 0.933 | | |
| Negative affect | | |  | |  | |  | |  | |  | | |  | | |  |  | | |  | | |
| Post-intervention | | | 0.00 | | -0.18, 0.17 | | 0.958 | | -0.12 | | -0.34, 0.11 | | | 0.301 | | | -0.48 | -1.86, 0.91 | | | 0.498 | | |
| 1-week follow-up | | | -0.14 | | -0.33, 0.05 | | 0.158 | | -0.04 | | -0.28, 0.19 | | | 0.712 | | | -0.26 | -1.74, 1.21 | | | 0.725 | | |
| 1-month follow-up | | | -0.17 | | -0.36, 0.03 | | 0.101 | | -0.03 | | -0.23, 0.18 | | | 0.805 | | | -0.38 | -1.60, 0.84 | | | 0.536 | | |
| Body image self-efficacy | | |  | |  | |  | |  | |  | | |  | | |  |  | | |  | | |
| Post-intervention | | | 2.15 | | -1.57, 5.87 | | 0.256 | | 0.13 | | -4.62, 4.88 | | | 0.959 | | | -2.84 | -32.93, 27.24 | | | 0.852 | | |
| 1-week follow-up | | | 3.14 | | -0.12, 6.39 | | 0.059 | | 0.02 | | -3.94, 3.98 | | | 0.992 | | | 0.38 | -24.28, 25.04 | | | 0.976 | | |
| 1-month follow-up | | | 3.89 | | 0.62, 7.16 | | 0.02 | | 0.94 | | -2.46, 4.34 | | | 0.588 | | | 1.64 | -18.72, 22.00 | | | 0.874 | | |
| Table S2  Analyses Testing whether Baseline Severity Moderates Intervention Effects | | | | | | | | | | | | | | | | | | | | | |  |  |
|  | | | **Difference in change score** | | | | | | | | |  | | |  | | | |  | | |  |  |
|  | | | **(Exp - Control)** | | | | | |  | | | **x Baseline DV scores** | | | | | | |  | | |  |  |
| **Outcome** | | | **Mean** | | **95% CIs** | | | | **p (two-tailed)** | | | **b** | | | **95% CIs** | | | | **p (two-tailed)** | | |  |  |
| Appearance positive | | |  | |  | | | |  | | |  | | |  | | | |  | | |  |  |
| Post-intervention | | | 0.29 | | 0.12, 0.47 | | | | 0.001 | | | -0.07 | | | -0.12, -0.02 | | | | 0.004 | | |  |  |
| 1-week follow-up | | | 0.3 | | 0.13, 0.47 | | | | 0.001 | | | -0.08 | | | -0.12, -0.03 | | | | 0.001 | | |  |  |
| 1-month follow-up | | | 0.57 | | 0.39, 0.76 | | | | <.001 | | | -0.14 | | | -0.19, -0.09 | | | | <.001 | | |  |  |
| Appearance negative | | |  | |  | | | |  | | |  | | |  | | | |  | | |  |  |
| Post-intervention | | | 0.22 | | 0.02, 0.42 | | | | 0.029 | | | -0.05 | | | -0.11, 0.01 | | | | 0.087 | | |  |  |
| 1-week follow-up | | | 0.30 | | 0.11, 0.48 | | | | 0.002 | | | -0.07 | | | -0.12, -0.02 | | | | 0.01 | | |  |  |
| 1-month follow-up | | | 0.41 | | 0.23, 0.58 | | | | <.001 | | | -0.10 | | | -0.15, -0.05 | | | | <.001 | | |  |  |
| Weight | | |  | |  | | | |  | | |  | | |  | | | |  | | |  |  |
| Post-intervention | | | 0.14 | | -0.3, 0.30 | | | | 0.111 | | | -0.04 | | | -0.09, 0.01 | | | | 0.086 | | |  |  |
| 1-week follow-up | | | 0.33 | | 0.14, 0.52 | | | | 0.001 | | | -0.09 | | | -0.15, -0.04 | | | | 0.001 | | |  |  |
| 1-month follow-up | | | 0.4 | | 0.22, 0.58 | | | | <.001 | | | -0.10 | | | -0.15, -0.05 | | | | <.001 | | |  |  |
| Positive affect | | |  | |  | | | |  | | |  | | |  | | | |  | | |  |  |
| Post-intervention | | | 0.08 | | -0.15, 0.31 | | | | 0.486 | | | -0.01 | | | -0.07, 0.05 | | | | 0.684 | | |  |  |
| 1-week follow-up | | | 0.15 | | -0.08, 0.38 | | | | 0.196 | | | -0.02 | | | -0.08, 0.04 | | | | 0.48 | | |  |  |
| 1-month follow-up | | | 0.41 | | 0.11, 0.71 | | | | 0.007 | | | -0.08 | | | -0.16, 0.00 | | | | 0.041 | | |  |  |
| Negative affect | | |  | |  | | | |  | | |  | | |  | | | |  | | |  |  |
| Post-intervention | | | 0.12 | | -0.04, 0.29 | | | | 0.14 | | | -0.06 | | | -0.13, 0.00 | | | | 0.055 | | |  |  |
| 1-week follow-up | | | 0.04 | | -0.17, 0.24 | | | | 0.735 | | | -0.05 | | | -0.12, 0.03 | | | | 0.247 | | |  |  |
| 1-month follow-up | | | 0.07 | | -0.15, 0.28 | | | | 0.533 | | | -0.06 | | | -0.14, 0.01 | | | | 0.098 | | |  |  |
| Body image self-efficacy | | |  | |  | | | |  | | |  | | |  | | | |  | | |  |  |
| Post-intervention | | | 4.51 | | 0.57, 8.45 | | | | 0.025 | | | -0.05 | | | -0.11, 0.00 | | | | 0.058 | | |  |  |
| 1-week follow-up | | | 6.84 | | 3.35, 10.32 | | | | <.001 | | | -0.08 | | | -0.13, -0.03 | | | | 0.001 | | |  |  |
| 1-month follow-up | | | 8.49 | | 4.81, 12.17 | | | | <.001 | | | -0.10 | | | -0.15, -0.05 | | | | <.001 | | |  |  |
| Table S3  Relationship between Engagement Metrics and Outcome Improvements | | | | | | | | | | | | | | | | | | | | |  |  |  |
| **Outcome** | **Engagement Variable** | | | | | | **Time-point** | | | | **b** | | | **95% CI** | | | | **p (two-tailed)** | | |  |  |  |
| Appearance positive | Completed at least one chatbot activity | | | | | | Post-intervention | | | | -0.02 | | | -0.20, 0.15 | | | | .778 | | |  |  |  |
|  |  | | | | | | 1 week follow-up | | | | -0.07 | | | -0.26, 0.12 | | | | .462 | | |  |  |  |
|  |  | | | | | | 1 month follow-up | | | | -0.07 | | | -0.25, 0.12 | | | | .48 | | |  |  |  |
|  | Number of chatbot activities completed | | | | | | Post-intervention | | | | 0.004 | | | -0.02, 0.03 | | | | .732 | | |  |  |  |
|  |  | | | | | | 1 week follow-up | | | | -0.01 | | | -0.03, 0.02 | | | | .622 | | |  |  |  |
|  |  | | | | | | 1 month follow-up | | | | -0.01 | | | -0.04, 0.02 | | | | .478 | | |  |  |  |
|  | Overall time spent on chatbot activities | | | | | | Post-intervention | | | | -0.001 | | | -0.01, 0.01 | | | | .782 | | |  |  |  |
|  |  | | | | | | 1 week follow-up | | | | -0.002 | | | -0.01, 0.005 | | | | .577 | | |  |  |  |
|  |  | | | | | | 1 month follow-up | | | | -0.002 | | | -0.01, 0.01 | | | | .579 | | |  |  |  |
|  | Average time spent on chatbot activities | | | | | | Post-intervention | | | | -0.001 | | | -0.01, 0.01 | | | | .815 | | |  |  |  |
|  |  | | | | | | 1 week follow-up | | | | -0.003 | | | -0.01, 0.01 | | | | .457 | | |  |  |  |
|  |  | | | | | | 1 month follow-up | | | | -0.003 | | | -0.01, 0.01 | | | | .56 | | |  |  |  |
| Appearance negative | Completed at least one chatbot activity | | | | | | Post-intervention | | | | 0.23 | | | 0.002, 0.47 | | | | .048 | | |  |  |  |
|  |  | | | | | | 1 week follow-up | | | | 0.23 | | | -0.01, 0.47 | | | | .056 | | |  |  |  |
|  |  | | | | | | 1 month follow-up | | | | 0.24 | | | -0.01, 0.48 | | | | .060 | | |  |  |  |
|  | Number of chatbot activities completed | | | | | | Post-intervention | | | | 0.02 | | | -0.01, 0.06 | | | | .174 | | |  |  |  |
|  |  | | | | | | 1 week follow-up | | | | 0.02 | | | -0.01, 0.06 | | | | .203 | | |  |  |  |
|  |  | | | | | | 1 month follow-up | | | | 0.03 | | | -0.01, 0.06 | | | | .174 | | |  |  |  |
|  | Overall time spent on chatbot activities | | | | | | Post-intervention | | | | 0.01 | | | -0.002, 0.01 | | | | .154 | | |  |  |  |
|  |  | | | | | | 1 week follow-up | | | | 0.01 | | | -0.003, 0.02 | | | | .177 | | |  |  |  |
|  |  | | | | | | 1 month follow-up | | | | 0.01 | | | -0.002, 0.02 | | | | .14 | | |  |  |  |
|  | Average time spent on chatbot activities | | | | | | Post-intervention | | | | 0.01 | | | -0.004, 0.02 | | | | .216 | | |  |  |  |
|  |  | | | | | | 1 week follow-up | | | | 0.01 | | | -0.004, 0.02 | | | | .238 | | |  |  |  |
|  |  | | | | | | 1 month follow-up | | | | 0.01 | | | -0.01, 0.02 | | | | .299 | | |  |  |  |
| Weight | Completed at least one chatbot activity | | | | | | Post-intervention | | | | 0.15 | | | -0.07, 0.38 | | | | .188 | | |  |  |  |
|  |  | | | | | | 1 week follow-up | | | | 0.15 | | | -0.10, 0.40 | | | | .249 | | |  |  |  |
|  |  | | | | | | 1 month follow-up | | | | 0.13 | | | -0.12, 0.38 | | | | .32 | | |  |  |  |
|  | Number of chatbot activities completed | | | | | | Post-intervention | | | | 0.02 | | | -0.01, 0.06 | | | | .171 | | |  |  |  |
|  |  | | | | | | 1 week follow-up | | | | 0.02 | | | -0.02, 0.06 | | | | .26 | | |  |  |  |
|  |  | | | | | | 1 month follow-up | | | | 0.02 | | | -0.02, 0.05 | | | | .445 | | |  |  |  |
|  | Overall time spent on chatbot activities | | | | | | Post-intervention | | | | 0.005 | | | -0.004, 0.01 | | | | .263 | | |  |  |  |
|  |  | | | | | | 1 week follow-up | | | | 0.01 | | | -0.005, 0.01 | | | | .302 | | |  |  |  |
|  |  | | | | | | 1 month follow-up | | | | 0.01 | | | -0.005, 0.01 | | | | .31 | | |  |  |  |
|  | Average time spent on chatbot activities | | | | | | Post-intervention | | | | 0.005 | | | -0.01, 0.02 | | | | .385 | | |  |  |  |
|  |  | | | | | | 1 week follow-up | | | | 0.004 | | | -0.01, 0.02 | | | | .523 | | |  |  |  |
|  |  | | | | | | 1 month follow-up | | | | 0.01 | | | -0.01, 0.02 | | | | .39 | | |  |  |  |
| Positive affect | Completed at least one chatbot activity | | | | | | Post-intervention | | | | 0.13 | | | -0.06-0.33 | | | | .178 | | |  |  |  |
|  |  | | | | | | 1 week follow-up | | | | 0.13 | | | -0.11-0.36 | | | | .295 | | |  |  |  |
|  |  | | | | | | 1 month follow-up | | | | 0.10 | | | -0.12-0.33 | | | | .360 | | |  |  |  |
|  | Number of chatbot activities completed | | | | | | Post-intervention | | | | 0.02 | | | -0.01-0.05 | | | | .183 | | |  |  |  |
|  |  | | | | | | 1 week follow-up | | | | 0.02 | | | -0.01-0.05 | | | | .262 | | |  |  |  |
|  |  | | | | | | 1 month follow-up | | | | 0.01 | | | -0.03-0.04 | | | | .763 | | |  |  |  |
|  | Overall time spent on chatbot activities | | | | | | Post-intervention | | | | 0.003 | | | -0.004, 0.01 | | | | .388 | | |  |  |  |
|  |  | | | | | | 1 week follow-up | | | | 0.004 | | | -0.004, 0.01 | | | | .317 | | |  |  |  |
|  |  | | | | | | 1 month follow-up | | | | 0.002 | | | -0.01, 0.01 | | | | .617 | | |  |  |  |
|  | Average time spent on chatbot activities | | | | | | Post-intervention | | | | 0.005 | | | -0.005, 0.01 | | | | .33 | | |  |  |  |
|  |  | | | | | | 1 week follow-up | | | | 0.01 | | | -0.004, 0.02 | | | | .236 | | |  |  |  |
|  |  | | | | | | 1 month follow-up | | | | 0.004 | | | -0.01, 0.01 | | | | .457 | | |  |  |  |
| Negative affect | Completed at least one chatbot activity | | | | | | Post-intervention | | | | -0.13 | | | -0.35, 0.09 | | | | .262 | | |  |  |  |
|  |  | | | | | | 1 week follow-up | | | | -0.16 | | | -0.39, 0.06 | | | | .151 | | |  |  |  |
|  |  | | | | | | 1 month follow-up | | | | -0.11 | | | -0.34, 0.12 | | | | .354 | | |  |  |  |
|  | Number of chatbot activities completed | | | | | | Post-intervention | | | | -0.02 | | | -0.05, 0.01 | | | | .209 | | |  |  |  |
|  |  | | | | | | 1 week follow-up | | | | -0.02 | | | -0.05, 0.01 | | | | .249 | | |  |  |  |
|  |  | | | | | | 1 month follow-up | | | | -0.01 | | | -0.05, 0.02 | | | | .502 | | |  |  |  |
|  | Overall time spent on chatbot activities | | | | | | Post-intervention | | | | -0.003 | | | -0.01, 0.01 | | | | .51 | | |  |  |  |
|  |  | | | | | | 1 week follow-up | | | | -0.01 | | | -0.02, 0.003 | | | | .185 | | |  |  |  |
|  |  | | | | | | 1 month follow-up | | | | -0.004 | | | -0.01, 0.01 | | | | .448 | | |  |  |  |
|  | Average time spent on chatbot activities | | | | | | Post-intervention | | | | -0.004 | | | -0.01, 0.004 | | | | .341 | | |  |  |  |
|  |  | | | | | | 1 week follow-up | | | | -0.01 | | | -0.01, 0.003 | | | | .18 | | |  |  |  |
|  |  | | | | | | 1 month follow-up | | | | -0.005 | | | -0.01, 0.004 | | | | .276 | | |  |  |  |
| Body image self-efficacy | Completed at least one chatbot activity | | | | | | Post-intervention | | | | -1.64 | | | -6.52-3.25 | | | | .510 | | |  |  |  |
|  |  | | | | | | 1 week follow-up | | | | -2.12 | | | -7.00-2.76 | | | | .394 | | |  |  |  |
|  |  | | | | | | 1 month follow-up | | | | -3.69 | | | -8.97-1.59 | | | | .170 | | |  |  |  |
|  | Number of chatbot activities completed | | | | | | Post-intervention | | | | 0.02 | | | -0.67-0.72 | | | | .949 | | |  |  |  |
|  |  | | | | | | 1 week follow-up | | | | 0.03 | | | -0.68-0.74 | | | | .941 | | |  |  |  |
|  |  | | | | | | 1 month follow-up | | | | -0.29 | | | -1.10-0.53 | | | | .490 | | |  |  |  |
|  | Overall time spent on chatbot activities | | | | | | Post-intervention | | | | -0.03 | | | -0.20-0.14 | | | | .753 | | |  |  |  |
|  |  | | | | | | 1 week follow-up | | | | -0.02 | | | -0.20-0.16 | | | | .856 | | |  |  |  |
|  |  | | | | | | 1 month follow-up | | | | -0.06 | | | -0.26-0.15 | | | | .590 | | |  |  |  |
|  | Average time spent on chatbot activities | | | | | | Post-intervention | | | | -0.02 | | | -0.24-0.20 | | | | .880 | | |  |  |  |
|  |  | | | | | | 1 week follow-up | | | | -0.02 | | | -0.24-0.20 | | | | .873 | | |  |  |  |
|  |  | | | | | | 1 month follow-up | | | | -0.05 | | | -0.31-0.21 | | | | .696 | | |  |  |  |
