# Supplementary material for: Using Chatbot Technology to Improve Brazilian Adolescents’ Body Image and Mental Health at Scale: Randomized Controlled Trial
Source: JMIR Mhealth Uhealth. 2023 Jun 19;11:e39934. doi: 10.2196/39934 (PMC10337468; doi:10.2196/39934)
Supplement: Multimedia Appendix 2 [file mhealth_v11i1e39934_app2.pdf]

CONSORT-EHEALTH Checklist V1.6.2 Report	Manuscript Number	39934

(based on CONSORT-EHEALTH V1.6), available at [http://tinyurl.com/consort-ehealth-v1-6].

Date completed
2/14/2023 8:35:48
by
Harriet Smith

Using chatbot technology to improve Brazilian adolescents' body image and mental health at-scale: A randomised controlled trial

TITLE

1a-i) Identify the mode of delivery in the title
"chatbot technology", a chatbot embedded in Facebook (Meta) Messenger

1a-ii) Non-web-based components or important co-interventions in title
Embedding the intervention into Facebook Messenger is key to our goal of large-scale dissemination and accessibility; thus, the intervention does not contain non-web-based components.

1a-iii) Primary condition or target group in the title
"Brazilian adolescents", specifically this intervention is aimed at 13-18 year olds.

ABSTRACT

1b-i) Key features/functionalities/components of the intervention and comparator in the METHODS section of the ABSTRACT
"body image chatbot containing micro-interventions" and "randomised into the chatbot or an assessment only control condition".

1b-ii) Level of human involvement in the METHODS section of the ABSTRACT
The chatbot is fully automated, as described in the main body of the manuscript.

1b-iii) Open vs. closed, web-based (self-assessment) vs. face-to-face assessments in the METHODS section of the ABSTRACT
"two-armed, fully remote, pre-registered randomised controlled trial" and participants "completed online self-assessments at baseline, post-intervention, 1-week and 1-month follow-up".

1b-iv) RESULTS section in abstract must contain use data
"...Brazilian adolescents aged 13-18 years (N = 1715; 52.5% girls) were randomised into the chatbot or an assessment only control condition". A majority of participants who entered the chatbot (79% [258 of 327]) completed one or more micro-intervention techniques, with participants completing an average of five techniques over the 72-hour intervention period. Chatbot users experienced small significant improvements in primary (state [Cohen's d = .30, [.25-.34] and trait body image [d range = .10 [.01–.18] – .26 [.13–.32]) and secondary outcomes (state [d =.28 [.22–.33] and trait positive affect [d range = .15 [.03–.27] – .23 [.08–.37], negative affect [d range = -.16 [-.30–-.02] – -.18 [-.33–-.03], self-efficacy [d range = .14 [.03–.25] – .19 [.08–.32]), relative to control. Intervention benefits were moderated by baseline levels of concerns (i.e., higher concerns experienced greater improvements), but not gender"

1b-v) CONCLUSIONS/DISCUSSION in abstract for negative trials
This is not a negative trial as there were improvements in the primary outcomes.

INTRODUCTION

2a-i) Problem and the type of system/solution
"8 in 10 young people in Brazil report body image concerns, with 1 in 5 reporting engagement in disordered eating and unhealthy weight control behaviours. Yet, to date, only two body image interventions have been evaluated among Brazilian populations and neither are widely available. They involve in-person implementation in group settings and therefore their sustainability is reliant on human and infrastructural resources....Micro-interventions are designed to be brief, digital and self-guided approaches that use in-the-moment techniques to provide immediate symptom reprieve or enhancement Chatbot technology offers an innovative pathway for engaging young people in evidence-based resources... prior research has been conducted in high income countries (e.g., Australia, China, Sweden and the US), and therefore the authors called for concerted research efforts in LMIC, stating there is a greater need for this technology in countries where shortage of mental health professionals is highest. To date, no chatbot has been developed for or tested among Brazilians".

2a-ii) Scientific background, rationale: What is known about the (type of) system
"Although the chatbots show promise as an effective and safe intervention modality; a majority target affective disorders, including anxiety and depression; a review heeded caution about their clinical significance, relative to treatment as usual and therefore concluded that greater research is needed to draw solid conclusions about this emerging service provision. More recently, micro-interventions have been developed and applied to young people (e.g., short films and online games), with these approaches proving acceptable and effective at eliciting immediate and short-term improvements in their body image and mood".

Does your paper address CONSORT subitem 2b?
"1: The chatbot was designed to provide immediate benefits to users. Adolescents will experience improvements in state-based body satisfaction and affect at the time of engagement with the chatbot. 2: Adolescents randomised into the intervention condition will experience greater improvements in trait-based body esteem, affect and body image self-efficacy at post-intervention, one-week and one-month follow-up, relative to the assessment only control condition. 3: Based on previous research...it was hypothesised that intervention effects will be moderated by gender, and baseline levels of body esteem, affect and body image self-efficacy. Specifically, intervention effects will be greatest among: girls; girls and boys with lower levels of trait body esteem; girls and boys with higher levels of trait negative affect and body image self-efficacy. 4: user engagement and adherence - given the novelty of this intervention, analyses will be of an exploratory nature. It is anticipated that greater engagement and adherence with the chatbot intervention will lead to greater improvements in trait-based outcomes".

METHODS

3a) CONSORT: Description of trial design (such as parallel, factorial) including allocation ratio
"two-arm fully remote pre-registered randomised controlled trial", "randomly assigned 1:1 to receive the chatbot intervention or an assessment only control. The randomisation scheme was generated by the research agency by means of a validated computer software. Blinding of participants was not possible due to the nature of the intervention"

3b) CONSORT: Important changes to methods after trial commencement (such as eligibility criteria), with reasons
"recruitment processes were heavily reliant on parents' literacy levels to provide informed consent, a video communicating the study information was embedded into the recruitment materials, implemented two weeks into recruitment following feedback from stakeholders. Further, the recruitment phase was extended from two weeks to 12 weeks, due to slow uptake." "This study was also conducted during COVID-19".

3b-i) Bug fixes, Downtimes, Content Changes
No changes or fixes were made during the trial.

4a) CONSORT: Eligibility criteria for participants
"Eligible participants were adolescents aged 13-18 years old who were Brazilian Portuguese speaking, a Brazilian resident, and had access to Facebook Messenger."

4a-i) Computer / Internet literacy
As the inclusion criteria stipulates that the participant must have access to Facebook Messenger and internet literacy is implied. Not necessary for it to be explicitly clarified.

4a-ii) Open vs. closed, web-based vs. face-to-face assessments:
"Recruitment was conducted via a Brazilian research agency (i.e., via email to their participant databases) and UNICEF's online communication platforms (e.g., U-Report; a free tool for community participation)... Following parental consent and participant assent, eligible participants completed pre-intervention self-assessments via a closed online survey (received via email), after which they were randomised into the intervention or control conditions. The online survey underwent user-testing before the trial began and the research agency monitored response rate and completion.... those in the intervention condition received a unique access link to the chatbot"

4a-iii) Information giving during recruitment
"Details of the study rationale and protocol have been published elsewhere. Prior to consent and assent, parents and participants were provided with an information sheet outlining the research procedures and associated risks, respectively. Consent withdrawal was possible at any time without cause for justification. Following parental consent and participant assent, eligible participants completed pre-intervention self-assessments via a closed online survey (received via email), after which they were randomised into the intervention or control conditions".

4b) CONSORT: Settings and locations where the data were collected
"Recruitment was conducted via a Brazilian research agency (i.e., via email to their participant databases) and UNICEF's online communication platforms"

4b-i) Report if outcomes were (self-)assessed through online questionnaires
"participants completed pre-intervention self-assessments via a closed online survey (received via email), after which they were randomised into the intervention or control conditions... At completion of the 72-hour intervention period, all participants were sent post-intervention surveys and again at the 1-week and 1-month time points. At the 1-month follow-up period, all participants were provided with contact details for accessible mental health support services in Brazil"

4b-ii) Report how institutional affiliations are displayed

5) CONSORT: Describe the interventions for each group with sufficient details to allow replication, including how and when they were actually administered

5-i) Mention names, credential, affiliations of the developers, sponsors, and owners

"JL is supported by the National Health and Medical Research Council Investigator Grant. PD is an independent consultant to the mental health policy and programming team at Instagram (owned by Meta, parent company of Facebook Messenger) and Dove (Unilever). The remaining authors declare that they have no competing interests." The development of the chatbot including stakeholder agreements and IP have been previously published in a protocol paper.		
**5-ii) Describe the history/development process**		
A pilot study was conducted with 154 Brazilian adolescents to assess Topity's user experience (e.g., ease of use) and acceptability (e.g., enjoyment; content relevance) prior to the main trial. Details published here: https://bmcpublichealth.biomedcentral.com/articles/10.1186/s12889-021-12129-1		
**5-iii) Revisions and updating**		
Topity was rated highly on overall user experience and acceptability across genders, thus no amendments were made to the intervention. However, it was frozen during the trial (i.e., only those given access via the trial could engage with the chatbot).		
**5-iv) Quality assurance methods**		
"This trial was pre-registered with ClinicalTrials.gov (NCT04825184)".		
**5-v) Ensure replicability by publishing the source code, and/or providing screenshots/screen-capture video, and/or providing flowcharts of the algorithms used**		
No source code was used. Included are screenshots of Topity included. Also see protocol paper for full details of the intervention content: https://bmcpublichealth.biomedcentral.com/articles/10.1186/s12889-021-12129-1.		
**5-vi) Digital preservation**		
https://www.facebook.com/topityunicef?mibextid=LQQJ4d		
**5-vii) Access**		
"intervention condition received a unique access link to the chatbot, and were encouraged to interact with the intervention as much as possible over the next 72-hour period. The unique access code was only compatible with the assigned user's Facebook Messenger; thus, ensuring confidential interactions between user and chatbot, as well as prohibiting non-participants from entering the chatbot during the trial period".		
**5-viii) Mode of delivery, features/functionalities/components of the intervention and comparator, and the theoretical framework**		
"Topity, is a body image chatbot hosted on Facebook Messenger, comprising of 8 micro-intervention techniques that address risk and protective factors for body image. Users can choose a host; a virtual young woman (Dandara) or man (Gabriel). The chatbot uses predefined rules and gamification to guide users through 8 micro-interventions; clustered into three themes (Family, Friends and Body image [2 techniques]; Social Media and Body Image [4 techniques]; Body Appreciation and Functionality [2 techniques]). The micro-interventions were informed by, and adapted from existing body image interventions that have been traditionally delivered in hard copy (e.g., self-help books) and/or in-person settings (e.g., individual therapy or group-based programs). The techniques teach users how to critically analyse and evaluate social media content to reduce vulnerability to negative influences (i.e., media literacy); identify and challenge unhelpful thinking styles and behaviours that perpetuate body image distress (i.e., cognitive behaviour theory for body image); and how to appreciate features and functions of the body beyond appearance (i.e., positive body image and embodiment theory). Each technique is 5-10 mins with a distinct beginning and end".		
**5-ix) Describe use parameters**		
Participants were asked to complete a minimum of one technique. After this, engagement with the intervention was ad libitum. "Once a technique was unlocked, there were no limits to the number of completions"		
**5-x) Clarify the level of human involvement**		
This is a stand alone self guided, digital intervention.		
**5-xi) Report any prompts/reminders used**		
"In the event participants did not engage with the bot after 12-, 16 and 23.5 hours, they received a Facebook Messenger notification encouraging participation".		
**5-xii) Describe any co-interventions (incl. training/support)**		
N/A. This is a stand-alone, self guided digital intervention. No co-interventions were provided.		
**6a) CONSORT: Completely defined pre-specified primary and secondary outcome measures, including how and when they were assessed**		
"primary outcome measures; the mean change in state (i.e., a single 11-point scale) and trait (i.e., Body Esteem Scale for Adolescents & Adults [BESAA] Brazil) body image. Specifically, the mean change in state body satisfaction between chatbot entry and post-technique, and the mean change in trait body esteem between pre-intervention and post-intervention. Secondary outcomes; the mean change in state affect (i.e., a single 11-point scale), trait positive and negative affect (i.e., Positive and Negative Affect Scale for Children 8-item), and self-efficacy in addressing body image concerns (i.e., The Body Image Self-Efficacy Scale). Treatment adherence (e.g., ≥ one micro-intervention technique) and user acceptability were also assessed at post-intervention".		
**6a-i) Online questionnaires: describe if they were validated for online use and apply CHERRIES items to describe how the questionnaires were designed/deployed**		
The CHERRIES checklist was guided the description of the questionnaire within the manuscript, however some of the details were N/A.		
**6a-ii) Describe whether and how "use" (including intensity of use/dosage) was defined/measured/monitored**		
Adherence was measured using various metrics monitored by the chatbot creators (Talk2U) and the research agency; including the number of people who accessed the bot, the average numbers of techniques completed, the average time spent in the bot, etc. Details can be found in the protocol paper: https://bmcpublichealth.biomedcentral.com/articles/10.1186/s12889-021-12129-1		
**6a-iii) Describe whether, how, and when qualitative feedback from participants was obtained**		
A pilot study was conducted prior to the main trial with the aim to gather qualitative feedback from adolescents as well as quantitative acceptability data. Details: https://bmcpublichealth.biomedcentral.com/articles/10.1186/s12889-021-12129-1		
**6b) CONSORT: Any changes to trial outcomes after the trial commenced, with reasons**		
"Recruitment was conducted via a Brazilian research agency (i.e., via email to their participant databases) and UNICEF's online communication platforms"		
**7a) CONSORT: How sample size was determined**		
**7a-i) Describe whether and how expected attrition was taken into account when calculating the sample size**		
Full details in the protocol paper under the 'statistical power' section: https://bmcpublichealth.biomedcentral.com/articles/10.1186/s12889-021-12129-1. Adopted a Monte Carlo simulation approach that tested power across a range of combinations of key statistical parameters for multilevel modelling: within-person sample size (varying from 3 to 5 estimates per person), intraclass correlation (ICC) for outcome (ranging from .3 to .6 in recognition that ICC values tend to be large for repeated measures designs), random slope variance (choosing .01, .09, and .25 to reflect small, moderate, and large amounts of variance), and effect size (setting at r = .2 to reflect small yet practically meaningful effect size). Attrition (~25%) was considered in the power analyses.		
**7b) CONSORT: When applicable, explanation of any interim analyses and stopping guidelines**		
"primary outcome measures; the mean change in state (i.e., a single 11-point scale) and trait (i.e., Body Esteem Scale for Adolescents & Adults [BESAA] Brazil) body image. Specifically, the mean change in state body satisfaction between chatbot entry and post-technique, and the mean change in trait body esteem between pre-intervention and post-intervention. Secondary outcomes; the mean change in state affect (i.e., a single 11-point scale), trait positive and negative affect (i.e., Positive and Negative Affect Scale for Children 8-item), and self-efficacy in addressing body image concerns (i.e., The Body Image Self-Efficacy Scale). Treatment adherence (e.g., ≥ one micro-intervention technique) and user acceptability were also assessed at post-intervention".		
**8a) CONSORT: Method used to generate the random allocation sequence**		
"Participants were randomly assigned 1:1 to receive the chatbot intervention or an assessment only control. The randomisation scheme was generated by the research agency by means of a validated computer software".		
**8b) CONSORT: Type of randomisation; details of any restriction (such as blocking and block size)**		
"1:1 randomisation to receive the intervention or assessment only control"		
**9) CONSORT: Mechanism used to implement the random allocation sequence (such as sequentially numbered containers), describing any steps taken to conceal the sequence until interventions were assigned**		
"Participants were randomly assigned 1:1 to receive the chatbot intervention or an assessment only control. The randomisation scheme was generated by the research agency by means of a validated computer software. The risk of bias from researchers was minimised due to having no contact with participants during the trial (i.e., recruitment; randomisation, survey dissemination and compensation was completed by the research agency)."		
**10) CONSORT: Who generated the random allocation sequence, who enrolled participants, and who assigned participants to interventions**		
"The randomisation scheme was generated by the research agency by means of a validated computer software."		
**11a) CONSORT: Blinding - If done, who was blinded after assignment to interventions (for example, participants, care providers, those assessing outcomes) and how**		
**11a-i) Specify who was blinded, and who wasn't**		
"Blinding of participants was not possible due to the nature of the intervention. Data analysts were blinded when conducting analyses on primary and secondary trait outcomes; however, this was not possible for primary and secondary state measures due to the within-group design."		
**11a-ii) Discuss e.g., whether participants knew which intervention was the "intervention of interest" and which one was the "comparator"**		
All participants were told they would get the opportunity to engage with a chatbot at some point due to the nature of the design and method (intervention vs assessment only control).		
**11b) CONSORT: If relevant, description of the similarity of interventions**		
N/A. Assessment only control received nothing whilst the intervention group engaged with the chatbot.		
**12a) CONSORT: Statistical methods used to compare groups for primary and secondary outcomes**		
Pre-specified in detail in the protocol paper: https://bmcpublichealth.biomedcentral.com/articles/10.1186/s12889-021-12129-1. "Linear mixed models were used to test hypotheses. For hyp 1, scores on state-based outcomes were regressed onto a time variable (coded 0 = pre-content, 1 = post-content) for the intervention arm only. For hyp 2-4, trait level outcome variables were regressed onto a time variable (dummy coded as baseline vs post-intervention, 1-week, and 1-month follow-ups) and group (0 = control, 1 = intervention) and an interaction between time and group to evaluate efficacy. Random effects were included for time, and an unstructured covariance matrix was used to estimate covariance among these random effects. Hyp's 3 and 4 included moderators for these random effects for time."		

**12a-i) Imputation techniques to deal with attrition / missing values**		
"missing data were handled using multiple imputation (m = 50) with chained equations, and participants were retained in the group they were assigned to at baseline, consistent with the principles of intention-to-treat"		
**12b) CONSORT: Methods for additional analyses, such as subgroup analyses and adjusted analyses**		
Baseline differences between intervention and control participants in terms of demographic factors were tested and full details of sub-group analyses are described. "Intervention benefits were moderated by baseline levels of concerns (i.e., higher concerns experienced greater improvements), but not gender."		
RESULTS		
**13a) CONSORT: For each group, the numbers of participants who were randomly assigned, received intended treatment, and were analysed for the primary outcome**		
As outlined in the flow diagram "figure 2"; 1,715 participants were randomised into the intervention (n = 858) or assessment only control (n = 857). Details of attrition and final numbers for analyses are included.		
**13b) CONSORT: For each group, losses and exclusions after randomisation, together with reasons**		
See "figure 2" within the manuscript.		
**13b-i) Attrition diagram**		
"Topity has reached >40,000 young people since the RCT commenced in April 2021." Attrition rates, adherence and completion rates are reported in the manuscript (during the trial period). But, we did not collect or evaluate data beyond the specific trial period.		
**14a) CONSORT: Dates defining the periods of recruitment and follow-up**		
"The study was a two-arm fully remote pre-registered randomised controlled trial conducted between 7th April and 8th August 2021 in Brazil." Follow-up points are determined by when the participants entered the chatbot (i.e., are phased, therefore no specific date can be reported).		
**14a-i) Indicate if critical "secular events" fell into the study period**		
N/A - no secular events occurred during the trial.		
**14b) CONSORT: Why the trial ended or was stopped (early)**		
The trial was extended due to slow recruitment uptake, then was executed as planned.		
**15) CONSORT: A table showing baseline demographic and clinical characteristics for each group**		
"Baseline characteristics of the sample are reported in Table 1"		
**15-i) Report demographics associated with digital divide issues**		
Demographics related to age, ethnicity and region within Brazil are "reported in Table 1". We included region as Brazil has both affluent urban areas and rural areas with restricted access to the Internet.		
**16a) CONSORT: For each group, number of participants (denominator) included in each analysis and whether the analysis was by original assigned groups**		
**16-i) Report multiple "denominators" and provide definitions**		
E.g., "Table 3 presents the Means, Standard Deviations, and Change Scores on Outcomes between Study Groups", Results revealed significant main effects of time for satisfaction (unstandardized coefficient, b = 0.60, 95% CI = 0.50-0.70, P<.001, d = .30) and affect (b = 0.51, 95% CI = 0.41-0.61, P<.001, d = .28), indicating that participants reported momentary improvements in these outcomes immediately following exposure to the chatbot micro-intervention techniques. Intervention adherence and user engagement, less than half (44%; 327 of 858 participants) of those randomised into the intervention utilised the chatbot. Of these, a majority (79%; 258 of 327 participants) completed the minimum intervention dose of one micro-intervention technique".		
**16-ii) Primary analysis should be intent-to-treat**		
Table 3 details the ITT analyses. "Hypothesis three tested whether mean differences on trait outcomes at each of the time-points were moderated by participant gender and baseline severity. Results from these analyses are presented in Supplementary Tables 1 and 2."		
**17a) CONSORT: For each primary and secondary outcome, results for each group, and the estimated effect size and its precision (such as 95% confidence interval)**		
Table 3 details the results for each group, the effect size and its precision (95% CI). "Results revealed significant main effects of time for satisfaction (unstandardized coefficient, b = 0.60, 95% CI = 0.50-0.70, P<.001, d = .30) and affect (b = 0.51, 95% CI = 0.41-0.61, P<.001, d = .28). At post-intervention, for the primary outcome of body esteem, significant mean differences were observed for the appearance positive (d = 0.13) and negative (d = 0.11) subscales, in favour of the intervention group experiencing improvements. A significant mean difference was not observed for the weight subscale (d = 0.01). At follow-up, the intervention group experienced greater improvements in these constructs, relative to the control group. Effect sizes were small, ranging from d = 0.10-0.26."		
**17a-i) Presentation of process outcomes such as metrics of use and intensity of use**		
"Hypothesis 4 tested whether there were any relationships between the four indices of chatbot usage and level of improvement in the primary and secondary outcomes. Results from these analyses are presented in the Supplementary Materials. We did not observe any significant relationships between levels of chatbot usage and the primary and secondary outcomes at the post-intervention and follow-up periods"		
**17b) CONSORT: For binary outcomes, presentation of both absolute and relative effect sizes is recommended**		
N/A - this RCT did not include binary outcomes.		
**18) CONSORT: Results of any other analyses performed, including subgroup analyses and adjusted analyses, distinguishing pre-specified from exploratory**		
"The [pre-specified] analyses revealed small significant improvements in primary (state [Cohen's d = .30, [.25-.34] and trait body image [d range = .10 [.01–.18] – .26 [.13–.32]) and secondary outcomes (state [d =.28 [.22–.33] and trait positive affect [d range = .15 [.03–.27] – .23 [.08–.37], negative affect [d range = -.16 [-.30–-.02] – -.18 [-.33—.03], self-efficacy [d range = .14 [.03–.25] – .19 [.08–.32]), relative to the control condition. Intervention benefits were moderated by baseline levels of concerns (i.e., higher concerns experienced greater improvements), but not gender". "User engagement and adherence analyses were of an exploratory nature".		
**18-i) Subgroup analysis of comparing only users**		
We did not perform sub-group analysis within the intervention group.		
**19) CONSORT: All important harms or unintended effects in each group**		
No harm to participants occurred and thus this was not indicated in the manuscript.		
**19-i) Include privacy breaches, technical problems**		
N/A. There were no real or perceived privacy breaches nor technical problems, thus these are not included in the manuscript.		
**19-ii) Include qualitative feedback from participants or observations from staff/researchers**		
This data is not available.		
DISCUSSION		
**20) CONSORT: Trial limitations, addressing sources of potential bias, imprecision, multiplicity of analyses**		
**20-i) Typical limitations in ehealth trials**		
To limit the impact of Type I error, we defined a primary outcome a-priori, and limited the inclusion of secondary outcomes.		
**21) CONSORT: Generalisability (external validity, applicability) of the trial findings**		
**21-i) Generalizability to other populations**		
The intervention, Topity was evaluated within the platform where it will be disseminated after the trial (i.e., Facebook Messenger), therefore the study has strong face validity. "The digital infrastructure developed for this chatbot can be implemented and disseminated in other countries, with content adapted for suitability in these contexts. This may include translating the content into different languages and incorporating examples that are appropriate and salient to that particular country and culture".		
**21-ii) Discuss if there were elements in the RCT that would be different in a routine application setting**		
Prompts via the chatbot are automated and would be used in real life, as per the study trial.		
**22) CONSORT: Interpretation consistent with results, balancing benefits and harms, and considering other relevant evidence**		
**22-i) Restate study questions and summarize the answers suggested by the data, starting with primary outcomes and process outcomes (use)**		
"Findings indicate this micro-intervention is an effective approach for eliciting small significant improvements in state- and trait-based outcomes for body image and associated well-being constructs. Girls and boys aged 13-18 years experienced small but significant improvements in state body satisfaction and positive affect immediately following engagement with Topity techniques. Users also reported small significant improvements in trait-based body esteem, positive and negative affect and body image self-efficacy, relative to the assessment only control condition. These group differences were observed at all three time-points for the appearance positive and negative subscales for the BESAA, and at 1-week and 1-month follow-up for positive and negative affect, and body image self-efficacy. Group differences on the weight subscale for the BESAA were only observed at 1-month, in favour of the intervention group experiencing improvements. Intervention effects were comparable across girls and boys, and therefore not moderated by gender. Effects were, however, moderated by baseline concerns with those reporting lower baseline body esteem, positive affect and self-efficacy experiencing greater intervention benefits than those with lower levels of concern. Lastly, no dose effects were observed (e.g., greater engagement was not associated with greater improvements)."		
**22-ii) Highlight unanswered new questions, suggest future research**		
"This trial provides further support for the adaptation of traditional prevention and intervention techniques (e.g., hard-copy and in-person) for use within digital environments. More broadly, mental health researchers are encouraged to examine existing evidence-based approaches and identify techniques that could be adapted for standalone use within digital environments. Isolating these techniques for self-guided use in digital settings that are already frequented by the consumer is likely to increase the accessibility, acceptability and scalability of mental health resources and in turn lead to impactful and sustainable change. Although micro-interventions are a promising intervention model, more research is needed in conceptualising how this model can be integrated into and enhance a stepped-care model for digital approaches targeting body and eating concerns. Given the growing literature into micro-interventions, a systematic review into this intervention model is both timely and necessary. Specifically, the review should provide a comprehensive overview of extant mental health micro-interventions. "		
Other information		

**23) CONSORT: Registration number and name of trial registry**		
Trial registration: Clinicaltrials.gov NCT00102401, http://clinicaltrials.gov/ct2/show/NCT00102401		
**24) CONSORT: Where the full trial protocol can be accessed, if available**		
Published protocol paper: https://bmcpublichealth.biomedcentral.com/articles/10.1186/s12889-021-12129-1		
**25) CONSORT: Sources of funding and other support (such as supply of drugs), role of funders**		
Funding: "Dove, Unilever"		
**X26-i) Comment on ethics committee approval**		
"This study received ethics approval from the Instituto Federal de Educação, Ciência e Tecnologia do Sudeste de Minas Gerais (4.232.804), Comissão Nacional de Ética em Pesquisa (4.582.466), and the University of the West of England (HAS 19.12.090). The study is registered with Clinical Trials.Gov (NCT04825184)"		
**x26-ii) Outline informed consent procedures**		
Full details of the consent processes are reported in the protocol paper: https://bmcpublichealth.biomedcentral.com/articles/10.1186/s12889-021-12129-1. After being screened as eligible, participants will be emailed an information pack to be read with their parent or guardian. Parents will provide consent as per the Brazilian General Data Protection Law, by uploading their signature to online identification verification software that is monitored by the research agency. "Parental consent and participant assent obtained via online communications".		
**X26-iii) Safety and security procedures**		
As detailed in the protocol paper, all information sheets outlined the benefits and/or risks of participating: https://bmcpublichealth.biomedcentral.com/articles/10.1186/s12889-021-12129-1.		
**X27-i) State the relation of the study team towards the system being evaluated**		
"Conflicts of interest: JL (APP1196948) is supported by the National Health and Medical Research Council Investigator Grant. PD is an independent consultant to the mental health policy and programming team at Instagram (owned by Meta, parent company of Facebook Messenger) and Dove (Unilever). The remaining authors declare that they have no competing interests".		
